# Immune cell involvement in brown adipose tissue functions

**DOI:** 10.1093/discim/kyac007

**Published:** 2022-10-31

**Authors:** Adeline Bertola, Alexandre Gallerand, Stoyan Ivanov

**Affiliations:** Université Côte d’Azur, CNRS, LP2M, Nice, France; Université Côte d’Azur, CNRS, LP2M, Nice, France; Université Côte d’Azur, CNRS, LP2M, Nice, France

**Keywords:** brown adipose tissue, immune cells, macrophages, thermogenesis

## Abstract

Brown adipose tissue (BAT) contains many immune cells. The presence of macrophages, monocytes, dendritic cells, T cells, B cells, and mast cells was documented in BAT. However, in comparison to white adipose tissue, relatively little is known on the impact of immune cells on BAT function. By directly interacting with BAT stromal cells, or by secreting pro- and anti-inflammatory mediators, immune cells modulate BAT activation and subsequently influence on adaptative thermogenesis and heat generation. In the current manuscript, we will focus on the diversity and functions of BAT immune cells.

## Graphical Abstract

**Graphical Abstract:**
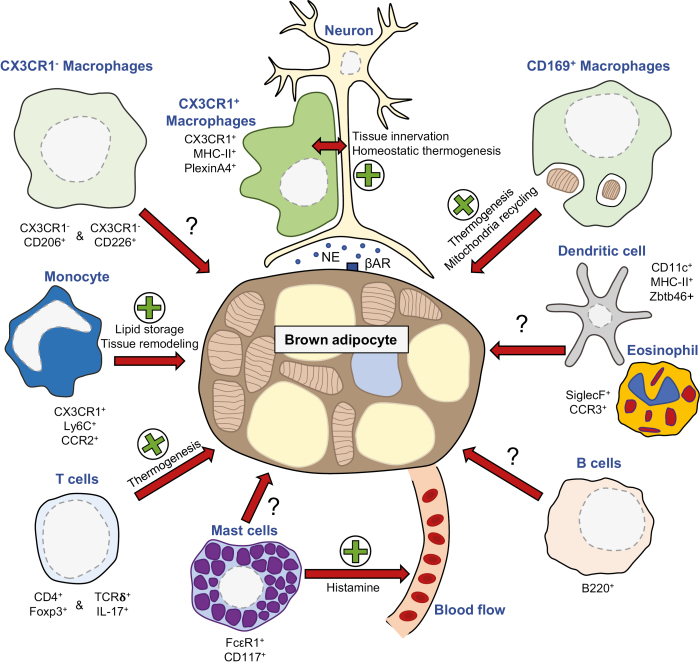

## Brown adipose tissue

Two main types of adipose tissue have been described in mammals: white adipose tissue (WAT) and brown adipose tissue (BAT), which have distinct developmental origin, anatomical location, morphology and functions. WAT is the main site for energy storage. White adipocytes accumulate excess calories in the form of triglycerides in a large unilocular lipid droplet and, when energy is needed, they hydrolyze them into fatty acids that are released into the blood circulation for use by other organs [1]. BAT, on the other hand, is specialized in expending energy through non-shivering thermogenesis, a process that plays a central role in the regulation of body temperature and whole-body energy homeostasis. Brown adipocytes possess numerous small multilocular lipid droplets and a high number of cristae-dense mitochondria expressing uncoupling protein 1 (UCP1) that allows the dissipation of energy in the form of heat at the expense of ATP synthesis [1,2]. Interestingly, UCP1-independent thermogenic mechanisms have also been described [3].

BAT is characterized by the presence of an extensive neurovascular network. BAT activity is mainly regulated by the sympathetic nervous system. Upon cold exposure, norepinephrine (NE) is released by sympathetic nerve endings and activates the canonical β-adrenergic receptor-cAMP-PKA pathway in brown adipocytes, resulting in the induction of UCP1 and other thermogenic genes as well as increased lipolysis and glucose uptake to support thermogenesis [4]. Adrenergic-independent activators of BAT have also been identified such as natriuretic peptides, bile acids, and FGF21 [5]. The generated heat is rapidly distributed to the rest of the body through the systemic blood circulation to maintain thermal homeostasis.

BAT is present in defined but dispersed areas in the body. In mice, the largest BAT depot is located in the interscapular area and additional smaller depots are found in the cervical, axillary, mediastinal, paravertebral, and perirenal regions [6]. Human newborns also have a prominent interscapular BAT depot that regresses with age. Considered absent in adult humans until relatively recently, functionally active BAT has been found in anatomical depots in the neck, shoulders, posterior thorax and abdomen, showing a high degree of topological and molecular similarities to the BAT depots of rodents [7–13]. In mice, increasing BAT activity reduces plasma triglyceride and cholesterol levels and attenuates diet-induced obesity, insulin resistance and atherosclerosis development [14,15] In humans, its presence is positively associated with cardiometabolic health [16,17]. Therefore, BAT has emerged as a potential therapeutic target against obesity and its associated metabolic disorders [18].

Besides classical brown adipocytes that reside in dedicated BAT depots, inducible thermogenic brown-like adipocytes, called beige or brite (brown-in-white) adipocytes have been discovered in WAT depots [19]. These cells emerge in response to stimuli such as cold exposure or pharmacological activation of β-adrenergic receptors. Although they share common morphological and biochemical characteristics with brown adipocytes, including multilocular lipid droplets and high UCP1 expression, beige adipocytes arise from different precursor cells and exhibit distinctive molecular profiles [20,21].

The stromal vascular fraction (SVF) of both WAT and BAT contains a variety of immune cells, including innate and adaptive immune cells. It is well established that immune cells in WAT regulate adipose tissue function and homeostasis through extensive cellular crosstalk [22]. Many studies reported a role for immune cells during adaptative thermogenesis but most of them focused only on the browning of subcutaneous WAT depots. For example, several groups established that eosinophils contribute to WAT browning, but these studies did not address whether and how eosinophils impact on the physiological activity of classical BAT [23–25]. In this context, features of immune cells involved in the regulation of WAT browning are often, mistakenly, also attributed to BAT resident immune cell populations. Therefore, here we decided to specifically review the current knowledge of the role of immune cells in classical BAT physiology and thermogenesis.

## Immune cell diversity in BAT

BAT contains multiple immune cell populations [26,27]. Macrophages, monocytes, dendritic cells, T cells, B cells, Natural Killer cells, and innate lymphoid cells were detected in healthy BAT. Housing animals at cold ambient temperature (4°C) induced BAT macrophage alternative polarization characterized by increased Arginase1 (Arg1), CD206 (Mrc1), and CD301 (Clec10a) expression [28]. Whether this is due to increased expression of these markers on the very same cells or monocyte recruitment leading to different cell composition remains to be determined. Importantly, during acute cold-exposure different mechanisms occurred in the thermogenic BAT and the subcutaneous beige adipose tissue. CCR2-dependent monocyte infiltration was instrumental to ensure optimal UCP1 expression in beige adipose tissue upon cold challenge [29]. However, in classical BAT, monocyte recruitment had no impact on UCP1 expression during cold exposure [29]. Initial studies suggested that adipose tissue macrophages are involved in thermogenesis by producing catecholamines [29]. Macrophage-specific tyrosine hydroxylase (Th) ablation (Lyz2^cre^ × Th^fl/fl^ mice) decreased core body temperature after cold exposure [29]. Importantly, the authors reported that norepinephrine levels were decreased in subcutaneous adipose tissue but not in BAT of Lyz2^cre^ × Th^fl/fl^ mice [29]. Recent study clearly established that adipose tissue macrophages do not express Th and do not produce detectable norepinephrine levels [30]. Furthermore, the authors demonstrated that macrophages do not contribute to subcutaneous adipose tissue beiging [30].

### Macrophages

**Figure 1:**
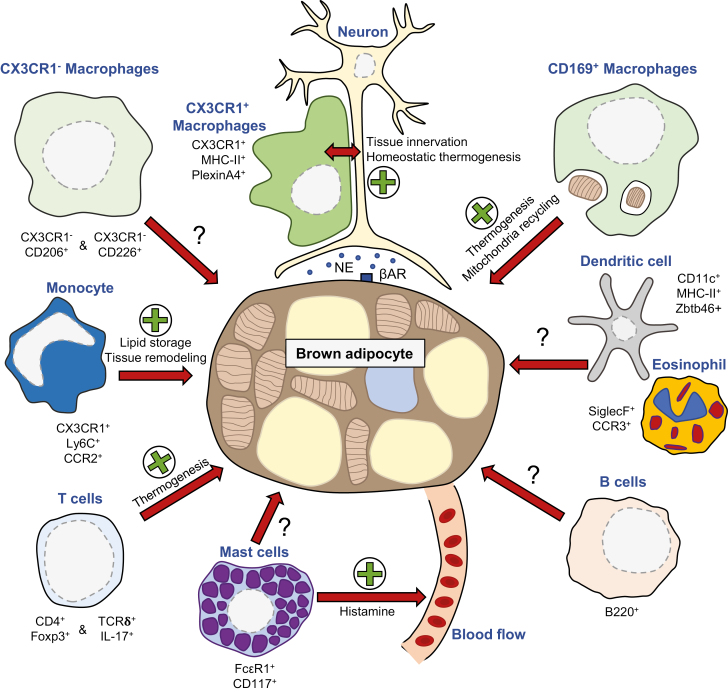
Schematic representation of BAT immune cell diversity and contribution to tissue homeostasis and adaptative thermogenesis.

Macrophages are characterized and identified by the co-expression of the markers CD64 and MerTK [31]. All tissue resident macrophages share this common signature which allows their separate analysis from other closely related myeloid cells including monocytes and dendritic cells (DCs). Brown adipose tissue macrophages are no exception to this rule [26]. Macrophage ontogeny and the tissue microenvironment imprint a particular signature to each organ residing macrophages [32–34]. The local tissue microenvironment induces a specific transcriptomic macrophage signature tightly associated with an organ-specific function [35,36]. The transcription factor controlling BAT macrophage differentiation is yet to be identified. Nevertheless, a specific role of BAT resident macrophages in the control of tissue innervation and thermogenesis was recently documented [37]. This function was mediated by a cluster of CX3CR1^+^ BAT macrophages expressing PlexinA4 (Figure 1) [37]. In BAT, populations of CX3CR1^+^ and CX3CR1^-^ macrophages co-exist at steady state [26,37]. The function of the CX3CR1^-^ BAT resident macrophages is yet to be defined. Of interest, two separate tissue resident macrophage subsets defined as Lyve1^low^CX3CR1^high^MHC-II^high^ and Lyve1^high^CX3CR1^low^MHC-II^low^ cells have been observed in multiple tissues (Figure 1) [38]. As described in BAT, the population of Lyve1^low^CX3CR1^high^MHC-II^high^ cells was located nearby nerve fibers [37,38]. In lung, skin and heart Lyve1^high^CX3CR1^low^MHC-II^low^ macrophages were observed in close proximity to blood vessels. The localization of Lyve1^high^ macrophages in BAT is to be established. We recently observed two additional tissue resident macrophages in BAT expressing relatively CD206 and CD226 (Figure 1) [26]. The ontology, localization, and functions of these cells require further investigation. The population of CD206^+^ BAT macrophages expressed genes associated with lipid metabolism [26]. Thus, one might expect that these cells are involved in handling local lipid content released from adipocytes at steady state or upon adrenergic stimulation. In contrast, CD226^+^ BAT macrophages were described by a transcriptomic signature consisting of multiple genes encoding for proteins involved in extracellular matrix remodeling [26]. These data pointed out toward a function in matrix shaping or stiffness. The development of novel experimental strategies to validate the aforementioned prediction is required to apprehend BAT macrophage subset function during homeostasis, cold exposure and thermoneutrality. Cold exposure triggered massive increase in BAT macrophage counts [39]. A recent report showed that housing mice in a cold environment (4°C) induced a higher CD206 expression on BAT macrophages in comparison to mice living at thermoneutrality (30°C) [40]. The authors demonstrated using macrophage depletion strategy (clodronate administration) that BAT macrophages ensure optimal thermogenesis by locally removing damaged mitochondria [40]. Importantly, the use of CD169-DTR mice further revealed that a subset of CD169-expressing macrophages mediates this specific function (Figure 1) [40]. The mechanisms triggering BAT macrophage local activation are yet to be fully defined. CXCL14 released by stromal cells promoted BAT macrophage accumulation and triggered their alternative polarization, thus contributing to adaptative thermogenesis [41]. Adipose tissue macrophages have been shown to internalize and degrade norepinephrine (NE) [42]. A subset of BAT macrophages that can degrade NE is yet to be identified. A specific population of macrophages, sympathetic neuron-associated macrophages (SAMs), express the membrane transporter Slc6a2 specialized in NE internalization [43]. BAT macrophages expressed Slc6a2 mRNA, and genetic ablation of this gene in immune cells increased local thermogenetic program and impacted on tissue architecture [43]. Whether BAT SAMs and the population of CX3CR1^+^ described by Wolf *et al.* are the very same cells is yet to be established.

### Monocytes

Monocytes are generated from bone marrow stem cells in a process named hematopoiesis. Following their entry in the blood circulation, two major monocyte subsets are identified in mice by their differential expression of the marker of unknown function Ly6C [44]. Ly6C^low^ patrolling monocytes have been shown to heal the blood vasculature [45,46], while Ly6C^high^, pro-inflammatory monocytes infiltrate peripheral tissues and give rise to tissue resident macrophages. Thus, Ly6C^high^ monocytes have been shown to play a crucial role during inflammatory diseases such as atherosclerosis and obesity. More recently, the presence of tissue monocytes outside the blood vasculature has been documented [47]. In tissues, monocytes express CD64 and F4/80, but not MerTK, which allows to differentiate them from neighbor macrophages [47]. Using this gating strategy, tissue monocytes have been identified in BAT and these cells have been shown to support tissue expansion (Figure 1) [26]. During BAT expansion, increased CCL2 expression likely account for monocyte recruitment [26]. Thermoneutrality, housing mice at 30°C, induced decreased monocyte BAT content in comparison to animals living at 22 and 4°C, environments shown to stimulate BAT activation [40,48]. The precise tissue localization of BAT monocytes remains to be defined. Importantly, whether these cells give rise to a particular BAT macrophage subset is yet to be clearly established. One could consider that this differentiation trajectory could be modulated during BAT stimulation by cold or stress and favor the generation of a particular BAT macrophage subset allowing to adapt to tissue demands. The environmental or metabolic cues influencing this differentiation are yet to be defined.

### Mast cells

Mast cells are heavily involved in allergic reactions and are the main source of histamine. FcεR1^+^CD117^+^ mast cells are present in mouse BAT (Figure 1) and toluidine blue staining of BAT sections showed that many mast cells adjoin microvessels [49,50]. Genetically induced deficiency of mast cells in *Kit*^W-sh/W-sh^ mice or their pharmacological stabilization using disodium cromoglycate or ketotifen improved obesity and glucose homeostasis upon Western diet feeding, in concert with higher energy expenditure and expression of UCP1 in BAT, indicating that mast cell may be linked to inhibition of thermogenesis in brown adipocytes [51]. In a subsequent study, Zhang *et al.* reported that functional inactivation of mast cells in mice on a chow diet increased systemic energy expenditure and thermogenesis; however, they did not detect higher UCP1 levels in BAT, suggesting differences in mast cell activity depending on the nutritional status [50].

Mast cells and histamine are enriched in BAT of aged mice [49]. Coculture of primary preadipocytes isolated from brown fat with the mast cell line p815 or treatment of differentiated brown adipocytes with conditioned media of p815 mast cells impaired brown adipogenesis, suggesting that BAT dysfunction during aging may be linked to mast cell activity [49].

Although the role of histamine in BAT remains to be clearly established, early studies indicated that histamine is involved in BAT thermogenic response in rats [52,53]. Rothwell *et al.* first reported that BAT contains substantial amounts of histamine [53]. Later on, using the potent mast cell degranulator Compound 48/80, Desautels *et al.* demonstrated that histamine is localized almost entirely within mast cells in BAT [52]. The histamine H2-receptor antagonist, cimetidine, blocked the increase in interscapular BAT temperature evoked by electrical stimulation of the ventromedial hypothalamic nucleus and reduced the effects of exogenously administered NE on metabolic rate, tissue blood flow and oxygen consumption of BAT [52,53]. Notably, histamine did not affect basal or NE-stimulated respiration in isolated brown adipocytes [52]. Thus, histamine acting through H2 receptors may play an important but indirect role in the thermogenic response of BAT.

### T cells

Several T-cell subsets have been involved in the regulation of BAT function in mice, including regulatory T (Treg) and unconventional γδ T cells (Figure 1). CD4^+^CD25^+^ Treg cells infiltrate BAT of female C57BL/6 mice and show a unique gene expression signature compared to splenic Treg cells and CD4^+^CD25^-^ conventional T cells in BAT and spleen [54]. Diphtheria toxin-induced systemic depletion of Treg cells in *Foxp3*^DTR^ mice impaired BAT activation in response to acute cold stimulation, as demonstrated by reduced oxygen consumption and heat production in concert with downregulation of thermogenic genes. In addition, Treg deficiency increased expression of pro-inflammatory markers and induced macrophage infiltration in BAT after cold exposure [54]. In line with this study, Kälin *et al.* reported that CD4^+^CD25^+^Foxp3^+^ Treg cells reside in BAT of male BALB/c mice [55]. Short-term cold exposure and β3-adrenergic receptor stimulation both enhanced Treg differentiation of BAT T cells *in vitro* and *in vivo*. Furthermore, Treg depletion using CD25 antibodies blunted the induction of the thermogenic gene program as well as β-oxidation- and lipolysis-related genes in BAT following β3-adrenergic receptor stimulation, while *Il6* levels increased. Conversely, *in vivo* transfer of CD25^+^Foxp3^+^ Treg cells enhanced BAT expression of thermogenic genes and markers of β-oxidation and lipolysis. Mechanistically, the authors demonstrated the role of a T-cell-specific Stat6/Pten axis in regulating Treg induction in BAT. Remarkably, Treg induction was impaired in BAT of UCP1-deficient mice, indicating a functional programming of BAT-residing T cells by thermogenesis [55]. Altogether these findings reveal the existence of a mutual crosstalk between Treg cells and BAT. Local BAT thermogenic activity supports Treg cell activation, which in turn is required for optimal BAT function. Interestingly, excessive insulin signaling in BAT Treg may contribute to diet-induced metabolic syndrome. Indeed, Treg-specific ablation of the insulin receptor (Insr) signaling in *Foxp3*^*cre*^*Insr*^*fl/fl*^ mice improved high fat diet-induced glucose tolerance and insulin sensitivity. These effects were associated with higher numbers and proportions of ST2^+^ Treg cells in BAT, concomitant with reduced inflammatory gene expression [56].

Similar to other adipose tissue depots, γδT cells are highly enriched in BAT compared with other organs. Although their number did not change after cold challenge, their frequency significantly increased [57]. Mice deficient in T-cell receptor (TCR) delta (*Tcrd*^−*/*−^), which lack γδT cells, are cold intolerant. Compared with wild-type littermates, their body temperature decreased more rapidly upon cold exposure and their BAT exhibited higher lipid content, less UCP1 protein and lower expression of thermogenic genes after acute cold challenge [57,58]. Kohlgruber *at al.* identified BAT γδT cells as a major source of IL-17A *ex vivo* and reported that *Il17a*^−*/*−^ mice phenocopied *Tcrd*^−*/*−^ mice, suggesting that γδT cells play a critical role in thermogenic control through IL-17A production [57]. In contrast, Hu *et al.* found that cold exposure upregulated *Il17f* mRNA expression in BAT, whereas that of *Il17a* was not detected [58]. Using a comprehensive set of mouse genetic models, including whole body deficiency of IL-17A, IL-17F, IL-17RA, IL-17RC, and adipocyte-specific knockouts of IL-17RC (i.e. *Adipoq*^*cre*^*Il17rc*^*fl/fl*^ and *Ucp1*^*cre*^*Il17rc*^*fl/fl*^ mice), they demonstrated that γδT cells promote adaptive thermogenesis by signaling to UCP1^+^ adipocytes via an IL-17F/IL-17RC axis, while IL-17A and IL-17RA were not involved [58]. Remarkably, sympathetic innervation of BAT was reduced in *Tcrd*^−*/*−^, *Il17f*^−*/*−^, and *Adipoq*^*cre*^*Il17rc*^*fl/fl*^ mice, implying that the thermogenic defect observed in these mice was related to impaired BAT innervation and that IL-17F/IL-17RC signaling pathway in adipocytes promotes growth of sympathetic axons in BAT. Further mechanistic studies identified TGFβ1 as the molecular link between the IL-17F/IL-17RC pathway and sympathetic nerve growth in BAT. Indeed, TGFβ1 expression was reduced in BAT from *Adipoq*^*cre*^*Il17rc*^*fl/fl*^ mice and restoration of its expression in brown adipocytes was sufficient to enhance BAT sympathetic innervation and cold tolerance in these mice. Inversely, TGFβ blocking sensitized mice to acute cold exposure [58]. Moreover, Vγ6^+^γδT cells represented a substantial fraction of the BAT-residing γδT cells. Notably, mice transgenic for a Vγ6^+^Vδ1^+^ TCR, in which Vγ6^+^Vδ1^+^ γδT cells are overrepresented, displayed enhanced sympathetic innervation of BAT and cold tolerance, thus highlighting the important role of this γδT cell subset. As expected, the adaptive thermogenesis-enhancing activity of Vγ6^+^Vδ1^+^ γδT cells was abrogated in *Adipoq*^*cre*^*Il17rc*^*fl/fl*^ mice [58]. Although the discrepancy between the above studies on the functional importance of the two IL-17 isoforms awaits further investigation, γδT cells appear to play a critical role in the regulation of BAT thermogenesis.

Interestingly, a recent study suggests that T-cell senescence may contribute to BAT dysfunction in aging. The proportion of CD3^+^ T cells is increased in the “whitened” BAT of 18-month-old mice [59]. These cells displayed senescent features, including loss of CD28 and expansion of CD44^+^CD62L^−^ memory T cells and a high percentage expressed IFN-γ. Coculture experiments using SVF cells from BAT and splenic CD3^+^ T cells isolated from old mice showed that senescent T-cell-derived IFN-γ inhibited preadipocyte-to-brown adipocyte differentiation [59].

### B cells

B220^+^ B cells are present in BAT in mice and their frequency is increased with diet-induced obesity whereas no change was observed in response to aging [60]. In general, there was a higher frequency of B220^+^ B cells in female mice compared with males [60]. Recent reports revealed that macrophages content in tissues follows a sex-specific pattern [61–63]. Whether this applies to BAT immune cells at steady state is yet to be determined. *muMT*^−*/*−^ (also known as *Ighm*^−*/*−^) mice, which lack B cells, have a normal cold tolerance [58]. Interestingly, it was reported that BAT has a markedly higher B-1 to B-2 cell ratio (≈0.2:1) than spleen and bone marrow (≈0.01:1) [64]. Further research is needed to characterize B cell subsets in BAT and their possible role in this tissue.

## Conclusion remarks

BAT emerged as an attractive new target to improve the metabolic status. BAT presence and activation are correlated with a better metabolic health in rodents and humans. In comparison to WAT, BAT is characterized by lower immune cell content. Nevertheless, the presence of innate and adaptative immune cells was revealed in BAT and these cells interfere with the key BAT function: thermogenesis. Thus, modulating BAT immune cell content or activation state could have a major impact on thermogenesis and energy expenditure, thus influencing tissue activity and impact on systemic metabolism by modulating glucose and fatty acid levels. BAT pharmacologic stimulation could improve the host metabolic status, nevertheless one should also consider eventual pitfalls such as impact on blood pressure, infection incidence and longevity. A careful investigation of the impact of BAT activation on systems physiology is required in pre-clinical models to better define the therapeutic benefits. Importantly, a better understanding of the functions of BAT immune cells might reveal new pathways controlling tissue activation and thermogenesis. To define BAT immune cell composition during aging and cold exposure is critical to apprehend whether immune cell fluctuations could be associated to and responsible for tissue function. A critical question is to determine immune cell composition in human BAT. To apprehend whether short-term cold exposure triggers metabolic benefits dependent on immune cell presence could help to improve strategies aiming at activating BAT thermogenesis and glucose consumption.

To date how BAT heat generation shapes the local and systemic immune response is yet to be clearly defined. Mild body temperature has been shown to modulate immune response during infections and tumor progression [65]. Importantly, BAT stimulation by cold exposure reduced tumor progression highlighting the therapeutical potential of BAT activation [66]. One needs to decipher whether BAT activation triggers higher glucose and fatty acid consumption thus limiting metabolite availability for highly proliferative tumor cells or eventually the heat generation activates locally the immune system.

Exploring BAT immune cell functions thus could help to develop and validate experimental strategies aiming to expand and activate BAT during metabolic diseases and cancer. Therefore, this might allow to lower systemic and local glucose availability and to eventually promote better metabolic state of the host and improve the disease outcome.

## Data Availability

Not applicable.

## Abbreviations

BAT: brown adipose tissue
WAT: white adipose tissue
UCP1: uncoupling protein 1
NE: norepinephrine
SVF: stromal vascular fraction
Th: tyrosine hydroxylase
SAMs: sympathetic neuron–associated macrophages
Treg: regulatory T cells
Insr: insulin receptor
TCR: T-cell receptor
